# Asymptomatic pseudoaneurysm 8 years after valve-sparing aortic root replacement

**DOI:** 10.1016/j.xjtc.2022.06.003

**Published:** 2022-06-16

**Authors:** Hiroaki Osada, Hideo Kanemitsu, Jiro Sakai, Motoyuki Kumagai, Kazuhiro Yamazaki, Kenji Minatoya

**Affiliations:** Department of Cardiovascular Surgery, Graduate School of Medicine, Kyoto University, Kyoto, Japan

**Figure:**
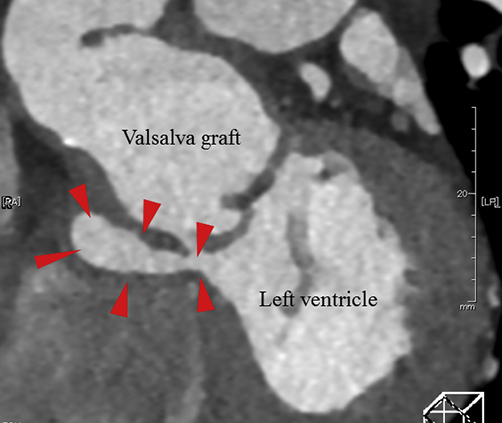
Pseudoaneurysm 8 years after VSRR located beneath the subannular stiches.

**Central Message**

We report a successful surgical repair of an LVOT pseudoaneurysm 8 years after VSRR for bicuspid aortic valve regurgitation and annuloaortic ectasia.

Valve-sparing aortic root replacement (VSRR) has been widely recognized as the optimal choice of the procedure for aortic root pathologies. Pseudoaneurysm formation after the procedure is a rare but a potentially devastating complication. We report a case of late complication of a left ventricular outflow tract (LVOT) pseudoaneurysm in a 54-year-old man, 8 years after VSRR. Ethics review is not required for case report implementation in our institution. Written informed consent was obtained from the patient for publication.

## Case Report

**Figure 1:**
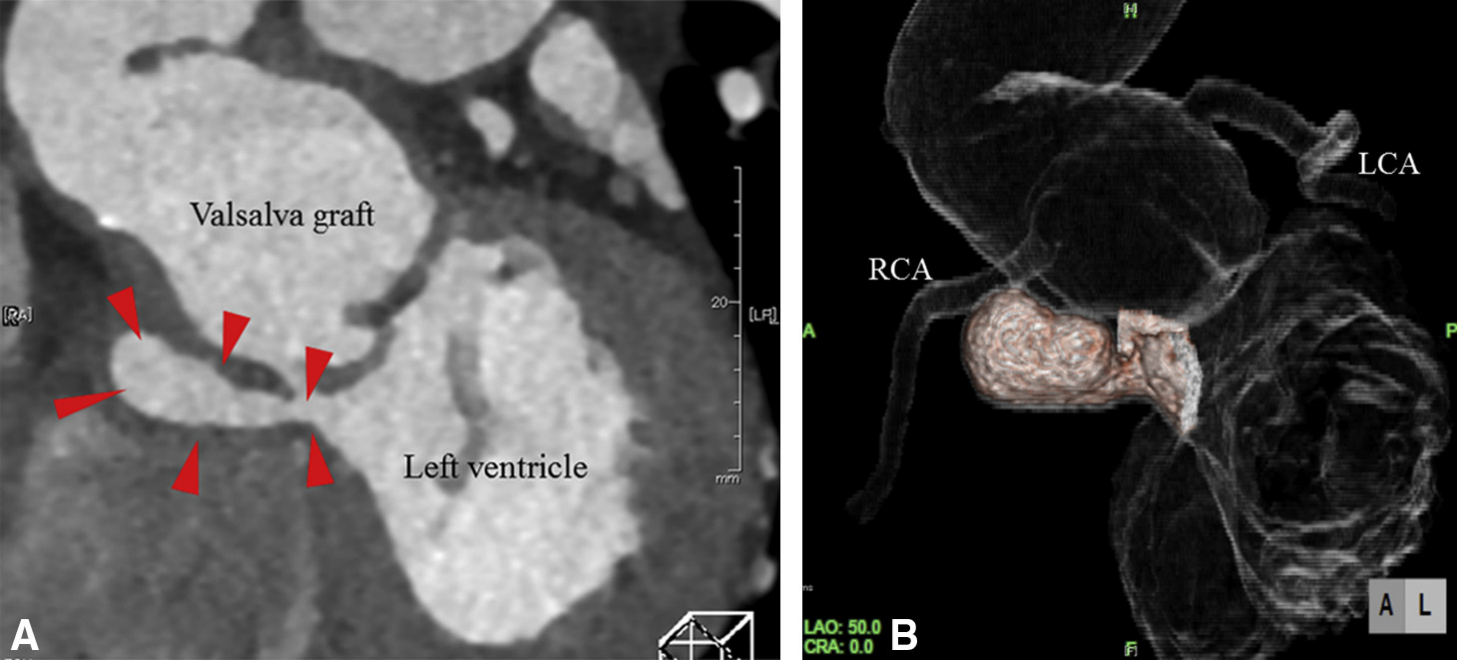
A, Preoperative contrast-enhanced computed tomography of the aortic root shows pseudoaneurysm formation (*red arrowhead*) from the left ventricle outflow tract. B, Preoperative 3-dimensional computed tomography of the aortic root. *RCA*, Right coronary artery; *LCA*, left coronary artery.

A 54-year-old man with a history the surgery of VSRR (David-V reimplantation) and hemiarch replacement for bicuspid aortic valve (Sievers type I) regurgitation and annuloaortic ectasia 8 years earlier, was referred to our department. On the most recent annual checkup via echocardiography, the patient had newly detected aortic root pseudoaneurysm without any symptoms. Enhanced chest computed tomography upon referral revealed a pseudoaneurysm formation around the right side of the aortic root, caudal side of the reconstructed right coronary ostium. The pseudoaneurysm seemed to be located from the LVOT through just below the Valsalva graft to the right atrial side (Figure 1, *A* and *B*). Other than the imaging tests, the patient showed no abnormal laboratory data.

At the initial surgery, measured diameter of the ventriculoaortic junction was 26 mm and a 28-mm Valsalva graft (Gelweave Valsalva Ante-Flo Gelatin Impregnated Woven Dacron Graft, Sultzer Vascutek) was used. Twelve 3–0 braided polyester with spaghetti horizontal mattress subannular stitches were placed and the Valsalva graft was seated. Remnant native tissue of the sinus of Valsalva was fixed with 5–0 polypropylene continuous suture. A redundant aortic valve was centrally plicated with 6–0 polypropylene, and left and right coronary ostium were reconstructed.

We initiated cardiopulmonary bypass with right femoral artery cannulation and bicaval venous drainage after resternotomy. For the better visualization, we employed a right atrium appendage incision toward the aortic root. We observed the LVOT through the transverse aortotomy using a dental mirror. The inflow of the pseudoaneurysm was located below the right noncommissure, the muscle portion just beneath the nondetached subannular stiches, which was the 7- to 8-mm slit parallel to the annulus. Because aortic regurgitation was well controlled before surgery, we tried preserving the structure without full root replacement, directly closed the inflow of the pseudoaneurysm with several stitches of pledgeted 4–0 polypropylene through the muscle portion of the LVOT to the Valsalva graft, and evaluated the closure using a thoracoscope.

**Figure 2:**
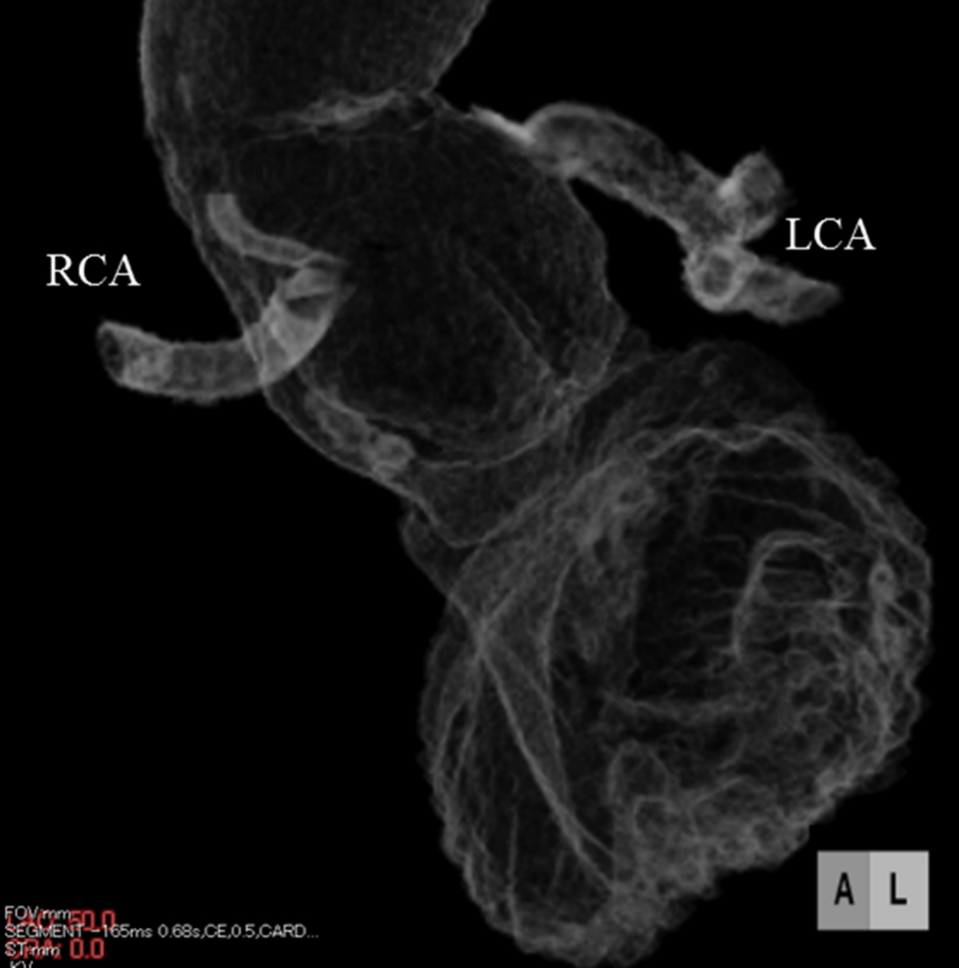
Postoperative 3-dimensional computed tomography of the aortic root revealed no residual pseudoaneurysm. *RCA*, Right coronary artery; *LCA*, left coronary artery.

The patient's postoperative course was uneventful other than transient atrioventricular block needed several days of temporary pacing. Postoperative enhanced chest computed tomography revealed favorable results without any extravasation from the aortic root (Figure 2). The patient was discharged after 2 weeks. Five months of outpatient follow-up were uneventful, and we are now carefully monitoring his progress.

## Discussion

VSRR has been widely recognized as the optimal choice of the procedure for the aortic root pathologies due to its durability and long term survival.^1^ Yamabe and colleagues^2^ reported 7 cases (0.8%) of aorta-related reintervention in a series of 882 cases of aortic root replacement, including Bentall procedure, most of which were associated with distal anastomosis pseudoaneurysm. Only a few case reports have been published previously about LVOT pseudoaneurysm accompanied by tearing of the subannular suture and direct or patch closure/total root replacement. They are associated with aortic wall abnormality such as connective tissue disorders, including Marfan syndrome and Loeys-Dietz syndrome.^3^, ^4^, ^5^ Uchida and colleagues^3^ discuss the etiology and the possibility of iatrogenic weakness of the LVOT induced by placing too many subannular stiches that are too tight.

In the present case, our patient, who had a bicuspid aortic valve, may have a similar condition. Intraoperative findings revealed nondetached subannular stitches and large slits at the LVOT. To our knowledge, there is no report of late complication pseudoaneurysm formation from LVOT, which is below the subannular stitches. Although the etiology is still unclear, the pseudoaneurysm could be controlled with direct closure with the preservation of the native aortic valve. It had been 8 years since the initial surgery and the complication could not have been predicted; thus, it is a reminder of the importance of regular checkups. Surgeons should be aware of this late complication.
